# Increased Krüppel-like factor 12 in recurrent implantation failure impairs endometrial decidualization by repressing Nur77 expression

**DOI:** 10.1186/s12958-017-0243-8

**Published:** 2017-03-31

**Authors:** Chenyang Huang, Yue Jiang, Jianjun Zhou, Qiang Yan, Ruiwei Jiang, Xi Cheng, Jun Xing, Lijun Ding, Jianxin Sun, Guijun Yan, Haixiang Sun

**Affiliations:** 1 Reproductive Medicine Center, The Affiliated Drum Tower Hospital of Nanjing University Medical School, Nanjing, 210008 People’s Republic of China; 2 Center for Translational Medicine, Department of Medicine, Thomas Jefferson University, Philadelphia, PA USA; 3 Collaborative Innovation Platform for Reproductive Biology and Technology of Nanjing University Medical School, Nanjing, 210008 People’s Republic of China

**Keywords:** KLF12, Nur77, Decidualization, Recurrent implantation failure

## Abstract

**Background:**

Decidualization is a prerequisite for successful implantation and the establishment of pregnancy. A critical role of impaired decidualization in subfertility has been established. In human endometrial stromal cells (hESCs), Krüppel-like factor 12 (KLF12) and Nur77 are novel regulators of decidualization. We investigated whether KLF12 impaired the decidualization of hESCs in recurrent implantation failure (RIF) patients.

**Methods:**

Endometrial tissues and hESCs were collected from RIF patients (*n* = 34) and fertile controls (*n* = 30) for in vitro analysis. Primary hESCs isolated from RIF endometrial tissues were used to evaluate the biological functions of KLF12 and Nur77. In addition, their molecular mechanisms were investigated by adenovirus-mediated overexpression. Gene expression regulation was examined by real-time-quantitative PCR (qRT-PCR), immunostaining and luciferase reporter assay. Further, blastocyst-like spheroid (BLS) and blastocyst implantation models were performed to examine the roles of KLF12 and Nur77 during embryo expansion on hESCs.

**Results:**

hESCs from the RIF patients showed a poor decidual response, mainly characterized by decreased decidual prolactin (dPRL) secretion, impaired transformation and limited BLS expansion. In addition, KLF12 expression was increased in endometrial tissues from the RIF patients compared with those from the fertile controls, especially in stromal compartments. The opposite results were observed for Nur77 expression in these tissues. KLF12 repressed hESC decidualization by decreasing Nur77 expression. Mechanistically, KLF12 bound to a conserved site in the Nur77 promoter region. Nur77 overexpression significantly reversed the KLF12-mediated repression of dPRL expression, decidual transformation and BLS/blastocyst expansion.

**Conclusions:**

KLF12 impairs endometrial decidualization by transcriptionally repressing Nur77, and Nur77 overexpression reverses the poor decidual response of hESCs in RIF patients.

**Electronic supplementary material:**

The online version of this article (doi:10.1186/s12958-017-0243-8) contains supplementary material, which is available to authorized users.

## Background

In human-assisted reproduction, the in vitro fertilization (IVF) success rate is strongly dependent on embryo implantation, which occurs in only 20–30% of patients after fresh embryo transfer. Moreover, approximately 10% of patients have unexplained recurrent implantation failure (RIF) after several IVF treatments [1]. At present, RIF caused by various etiological factors remains a challenge for fertility clinicians using assisted reproductive technology (ART) worldwide [2]. Embryo implantation involves a complex series of events that results in establishment of a connection between maternal and embryonic sections. Once the blastocyst emerges from the zona pellucida, it becomes juxtaposed and adheres to the luminal epithelium. Subsequently, the embryo expands and invades the endometrial stroma [3, 4].

At the time of implantation, a crucial event occurs in which human endometrial stromal cells (hESCs) undergo transformation in a process termed decidualization [5]. The adequate decidualization of hESCs is a precondition for embryo invasion and the maintenance of pregnancy [6]. Decidual cells coordinate the process of trophoblast invasion and serve to protect the embryo from maternally derived insults [7]. The decidualization process is defined by the mesenchymal-to-epithelial transformation of endometrial fibroblasts and stromal cells into secretory epithelioid decidual cells [8]. One of the most characteristic features of decidualization is the secretion of decidual prolactin (dPRL), which is a decidual-specific protein [9]. Decidual prolactin, a crucial ‘endocrine’ and ‘autocrine/paracrine’ cytokine secreted by the decidualized endometrium, is believed to regulate many functions in embryo implantation. This cytokine controls multiple important processes in early pregnancy, including the proliferation and differentiation of epithelial cells and hESCs, trophoblast cell growth, angiogenesis and immune regulation [10]. Impaired decidualization and decreased dPRL secretion have been described in a number of diseases, including endometriosis, adenomyosis, recurrent pregnancy loss (RPL), and implantation failure [11–14]. Several key factors, such as FOXO1A, Krüppel-like factor 12 (KLF12) and orphan nuclear receptor Nur77, have been implicated in regulation of the decidualization process [15–17]. We have previously demonstrated that KLF12, a progestogen target gene, is expressed in the glandular epithelium (GE) and in stromal cells of secretory phase endometrial tissue and that it negatively regulates endometrial decidualization through the transcriptional repression of target genes both in vitro and in vivo [18]. This study has revealed that the excessive expression of KLF12 and decreased expression of Nur77 in the endometrium of RIF patients.

However, to elucidate the exact mechanism of the interaction between KLF12 and Nur77, we visually scanned the wild-type Nur77 sequence (−3000 to +100 relative to the transcription start site) and found a specific KLF12 binding site in the promoter. Therefore we hypothesized that in patients with RIF enhanced KLF12 expression impairs endometrial decidualization by transcriptionally repressing Nur77.

## Methods

### Patient sample collection

Endometrial samples were obtained via endometrial biopsy between days 19 and 23 of the menstrual cycle from all participants, including 30 fertile women and 34 patients with RIF. Among these, the endometrial tissues from 18 fertile women and 22 RIF patients were used for samples investigation and others were used for hESCs isolation. This study was conducted at Drum Tower Hospital from January 2014 to December 2015. None of the patients received hormonal therapy during the 3 months prior to surgery, and they all exhibited menstrual regularity, with 26–33 day cycles. The details of these patients are summarized in Table 1. Recurrent implantation failure was defined as failure to achieve pregnancy following a minimum of three fresh or frozen cycles, during which at least four good-quality embryos were transferred to the uterus [19]. Patients who had experienced at least one normal pregnancy and/or delivery were recruited as fertile controls. Women with polycystic ovarian syndrome (PCOS), hydrosalpinx, endometriosis, adenomyosis, endometrial hyperplasia or endometrial polyps were not included. This study was approved by the Institutional Review Board of Nanjing Drum Tower Hospital on 5 December, 2013 (2013-081-01).

**Table 1:** Demographic details of the participants in this study

Fertile	FER (*n* = 30)	RIF (*n* = 34)	P
Age(years)	30.3 ± 5.1	31.2 ± 3.4	ns
Body mass index(kg/m^2^)	21.5 ± 2.4	22.3 ± 3.1	ns
Menstrual cycle(days)	30.0 ± 6.1	33 ± 10.9	ns
Endometrial thickness(mm)	10.5 ± 1.7	9.6 ± 1.6	ns
P_4_ on hCG day (pg/ml)	9.2 ± 3.7	9.6 ± 3.1	ns
No. of transferred embryos	1.97 ± 0.2	8.1 ± 3.6	s

The data are presented as the mean ± SD unless otherwise indicated. P_4_: Serum progesterone concentration

### Isolation of hESCs and decidualization in vitro [20]

Human endometrial stromal cells were isolated from mid-secretory phase endometrial tissues collected from fertile control and RIF patients in accordance with the standards described above. These cells were isolated and cultured as previously described. To induce decidualization, the hESCs were cultured in phenol red-free DMEM/F12 medium (HyClone, Thermo Scientific, South Logan, UT, USA) containing 2.5% (v/v) charcoal/dextran-treated fetal bovine serum (FBS; HyClone, Thermo Scientific, South Logan, UT, USA), 100 IU/ml penicillin, and 100 μg/ml streptomycin supplemented with 0.5 mM 8-Br-cAMP and 1 μM medroxyprogesterone-acetate (MPA) (Sigma, St. Louis, MO, USA) for the indicated durations.

### Construction of adenoviruses

Adenoviruses harboring the full-length KLF12 (Ad-Flag-KLF12, NCBI Reference Sequence: NM_007249.4) and Nur77 genes (Ad-Flag-Nur77, NCBI Reference Sequence: NM_173157.1) were generated using an AdMax (Microbix Biosystems, Inc., Toronto, Canada) system, according to the manufacturer’s recommendations. The viruses were packaged and amplified in HEK293A cells and purified by CsCl banding.

### Blastocyst-like spheroid (BLS) implantation model

As the BLS implantation model has been demonstrated to be an accurate and effective in vitro assay for use in embryo implantation research, it was employed in these studies, with modifications [21, 22]. Briefly, BeWo cells were detached with 0.25% trypsin (Gibco BRL/Invitrogen, Carlsbad, CA, USA) after they had reached 80% confluence. The BeWo cell suspensions were then placed in 35 mm^2^ dishes coated with an anti-adhesive polymer, poly-2-hydroxyethyl methacrylate (poly-HEMA; Sigma, St. Louis, MO, USA), to induce the formation of BLSs with diameters ranging from 150 to 200 μm after 48 h of culturing. We cocultured BLSs with hESCs to generate an in vitro model of embryo implantation. Confluent monolayer hESCs were decidualized for 3 days before being exposed to BLSs in 24-well culture plates as indicated in each figure legend. After substandard BLSs were removed using a 0.15 mm filter, several BLSs were transferred per chamber onto the confluent monolayer hESCs under a dissection microscope. The cocultures were maintained at 37 °C in a humidified atmosphere with 5% CO_2_ for 2 days. The attached and expanded BLSs were photographed at the indicated time points, and the area of expansion was expressed as a fold increase normalized to that of the untreated group. All cocultures were monitored using a microscope (Leica, Wetzlar, Germany).

### Mouse blastocyst implantation model [23]

All experimental procedures involving animals were performed in accordance with the guidelines of the Experimental Animals Management Committee (Jiangsu Province, China) and were approved by the Institutional Animal Care and Use Committee of Nanjing Drum Tower Hospital (SYXK 2014–0052). Mouse blastocysts were collected from the uterus of a pregnant mouse at 3.5 days post-coitus (dpc) and then transferred onto hESCs in 4-well plates, with three blastocysts per well. Next, the blastocysts and decidual hESCs were cocultured for 2 days. The attached blastocysts and their outgrowth were photographed at the indicated time points. All cocultures were monitored using a microscope (Leica, Wetzlar, Germany).

### RNA isolation and quantitative real-time PCR (qRT-PCR)

Total RNA was extracted from hESCs using Trizol reagent (Invitrogen, Carlsbad, CA, USA), according to the manufacturer’s instructions. One microgram of total RNA was reverse transcribed in a total volume of 20 μl. Reverse transcription was performed using random primers, and qRT-PCR was conducted with a MyiQ Single-Color Real-Time PCR Detection System (Bio-Rad, Hercules, CA, USA). The following primers were also used for the indicated genes: KLF12, 5′-CCTTTCCATAGCCAGAGCAG-3′ and 5′-TTGCATCCCTCAAAATCACA-3′; Nur77, 5′-ACCCACTTCTCCACACCTTG-3′ and 5′-ACTTGGCGTTTTTCTGCACT-3′; PRL, 5′-CACTACATCCATAACCTCTC-3′ and 5′-ATGCTGACTATCAAGCTCAG-3′; and 18S rRNA, 5′-CGGCTACCACATCCAAGGAA-3′ and 5′-CTGGAATTACCGCGGCT-3′ [14, 18]. Reactions were run in duplicate using RNA samples from three independent experiments. The fold change in expression of each gene was calculated using the 2^-△△CT^ method, with 18S rRNA as an internal control.

### Western blotting

Proteins were extracted as described previously [17]. The protein concentrations were measured by Bradford assay (Bio-Rad, Hercules, CA, USA). Equal amounts (25 μg) of protein were separated on a 10% SDS-polyacrylamide gel and transferred onto polyvinylidene fluoride membranes (Millipore, Billerica, MA, USA). Immunoblotting was performed by incubating the membranes with primary antibodies against KLF12 (1:2000; sc-84347, rabbit Polyclonal Antibody, Santa Cruz Biotechnology, Santa Cruz, CA, USA), Nur77 (1:1000; 3960, rabbit Monoclonal Antibody, Cell Signaling Technology, Danvers, MA, USA) and GAPDH (1:10000; AP0063, GAPDH polyclonal antibody, Bioworld Technology, MN, USA), followed by incubation with a goat anti-rabbit horseradish peroxidase (HRP)-conjugated secondary antibody (1:10000; BS13278, Bioworld Technology, St. Louis Park, MN, USA) and Flag-HRP (1:5000; A8592, Sigma, St. Louis, MO, USA). Detection was performed using an enhanced chemiluminescence kit (Amersham Biosciences Corp., Piscataway, NJ, USA), and densitometric analysis of each band was performed with Quantity-one (Bio-Rad, Hercules, CA, USA) software.

### Immunostaining

Formalin-fixed, paraffin-embedded uterine endometria were serially sectioned, dewaxed with xylene and rehydrated through a graded alcohol series, and then endogenous peroxidase activity was blocked using freshly prepared phosphate-buffered saline (PBS) containing 3% hydrogen peroxide for 20 min. Antigen retrieval was conducted by autoclaving the samples at 121 °C for 15 min in the presence of EDTA (pH 9.0), followed by incubation in blocking solution for 30 min. Next, the sections were washed with PBS and incubated with antibodies against KLF12 (1:600; sc-84347, rabbit Polyclonal Antibody, Santa Cruz Biotechnology, Santa Cruz, CA, USA) and Nur77 (1:250; 3960, rabbit Monoclonal Antibody, Cell Signaling Technology, Danvers, MA, USA) overnight at 4 °C. Subsequently, the sections were rinsed with PBS and incubated with an HRP-conjugated goat anti-rabbit secondary antibody at 37 °C for 20 min. HRP activity was detected using diaminobenzidine (Invitrogen, Carlsbad, CA, USA), and the sections were counterstained with hematoxylin. Control sections were run concurrently with the experimental sections using nonspecific rabbit IgG, and they were similarly pretreated. Nonspecific staining was not detected in the controls [18].

### Immunofluorescence staining for F-actin filaments

hESCs grown in 8-well chambers (Millipore, Billerica, MA, USA) were exposed to a decidualization stimulus of 8-Br-cAMP plus MPA for the indicated durations and then fixed with 4% paraformaldehyde (w/v) for 30 min at room temperature. Next, the cells were washed with PBS and permeabilized with 0.5% Triton X-100 in PBS at room temperature. Subsequently, the cells were blocked with 3% BSA in PBS and incubated with fluorescein isothiocyanate-labeled phalloidin (1:300; P5282, Sigma, St. Louis, MO, USA) at 4 °C overnight. Cell nuclei were stained with DAPI (5 μg/ml) on the following day. Finally, the cells were visualized using a confocal microscope (Leica, Wetzlar, Germany) [24].

### Chromatin immunoprecipitation (ChIP)/PCR assay

hESCs (70% confluence) were infected with Ad-LacZ and Ad-Flag-KLF12 (at a multiplicity of infection (MOI) of 20) for 24 h and then maintained in phenol red-free DMEM/F12 medium containing 2.5% charcoal/dextran-treated FBS with 0.5 mM 8-Br-cAMP plus 1 μM MPA. After 48 h, the hESCs were prepared for ChIP using Flag beads as described previously [25]. The recovered DNA was analyzed by PCR and real-time PCR. Fold changes were calculated as 2^-ΔΔCT^ values and are presented relative to LacZ (after normalization to the input DNA). The PCR mixtures contained 2 μl DNA, standard PCR reagents, and 50 pmol of each primer (Nur77 5′-GTTGAGAACTGGGTGGGTGG-3′ and 5′-CCTTCCACCCTGATCTCTCC-3′ (spanning 200 bp), specific for Nur77 promoter DNA fragments). A negative control primer was set targeting a sequence distal (−7030 to −6864 bp).

### Avidin-biotin conjugate DNA precipitation (ABCD) assay

Double-stranded oligonucleotides were designed based on the Nur77 promoter (promoter ID: 8941) sequence (−1391 to −1357 bp). The 5′ end of the sense strand was biotinylated, and a deletion and a mutation were introduced (deletion and mutation of the CAGTGGG sequence) to remove the specific binding site for KLF12. The following primers were designed: human Nur77 wild type: 5′-biotin-ATGGGGGTCGCAGTGGGGTGGCAGGGCTCTCTTTT-3′; human Nur77 wild-type reverse:5′-AAAAGAGAGCCCTGCCACCCCACTGCGACCCCCAT-3′; human Nur77 del: 5′-biotin-ATGGGGGTCGGTGGCAGGGCTCTCTTTT-3′; human Nur77 del reverse: 5′-AAAAGAGAGCCCTGCCACCGACCCCCAT-3′ and human Nur77 mut: 5′-biotin-ATGGGGGTCGCACAAAGGTGGCAGGGCTCTCTTTT-3′; human Nur77 mut reverse:5′-AAAAGAGAGCCCTGCCACCTTTGTGCGACCCCCAT-3′. hESCs were infected with Ad-LacZ and Ad-Flag-KLF12 (MOI = 20) for 24 h and then incubated with 8-Br-cAMP and MPA for an additional 48 h. Cell extracts were harvested and lysed in RIPA buffer. Each double-stranded DNA sample (500 pmol) was incubated with 600 μg of cell extract at 4 °C for 4 h, and the protein complexes were pulled down using streptavidin agarose beads (Sigma, St. Louis, MO, USA) in binding buffer (10 mM Tris, pH 8.0, 150 mM NaCl, 0.5% Triton X-100, 0.5 mM DTT, 0.5 mM EDTA, 10% glycerol, and 20 μg/ml poly [dI–dC]) containing a protease inhibitor cocktail. The beads were washed four times with the same buffer. Proteins were resolved by SDS-PAGE and then electro transferred onto a polyvinylidene fluoride membrane. Probing was performed with a Flag-HRP antibody (1:5000; A8592, Sigma, St. Louis, MO, USA). Immunodetection was accomplished by enhanced chemiluminescence (Millipore, Billerica, MA, USA) [14].

### Luciferase reporter assay

The wild-type Nur77 promoter sequence, which spans from −1604 to +98 bp relative to the transcription start site, was amplified by PCR from hESC genomic DNA using the following primers: 5′-CGGGGTACCTGTGGGACCTTTGAGTGGGC-3′ and 5′-CCGCTCGAGGACTGGCGCCCCGAGTCTCA-3′. In addition, we designed the following primers for the deleted Nur77 promoter sequence from −710 to +98 bp: 5′-CGGGGTACCGGGGCAGCCTCTCAGCCTGA-3′ and 5′-CCGCTCGAGGACTGGCGCCCCGAGTCTCA-3′. The PCR products were cloned into pGL3-basic luciferase reporter plasmids (Promega, Madison, WI, USA). Preconfluent (70%) hESCs in 12-well plates were infected with Ad-Flag-KLF12 and then transfected with 300 ng of the luciferase reporter plasmids using Lipofectamine 2000 (Life Technologies,Carlsbad, CA, USA) for 48 h. Cell lysates were assayed for luciferase activity using a Luciferase Assay System (Promega, Madison, WI, USA), and the activity was measured using a luminescence counter (Centro XS3 LB 960, Berthold Technologies) [18].

### Prolactin measurement by enzyme-linked fluorescent assay (ELFA)

Prolactin levels were measured using a Mini-Vidas V.B 02.96 system with a Vidas Prolactin Kit (bioMerieux, France). The lower limit of detection using the kit was 0.5 ng/ml.

### Statistical analysis

The data are presented as the mean ± SEM. All experiments were performed at least three times. Student’s t-test was used for comparisons between two groups. Statistical analysis was conducted by ANOVA, followed by the Student–Newman–Keuls test, for experiments involving more than two groups. In addition, statistical analysis for the dPRL level of RIF hESCs and the area of BLS expansion at indicated time points was conducted by repeated measures data ANOVA. Pearson correlation analysis was used to assess the relationship between KLF12 and Nur77. P values of less than 0.05 were considered statistically significant.

## Results

### Impaired decidualization of hESCs from RIF patients

**Fig. 1:**
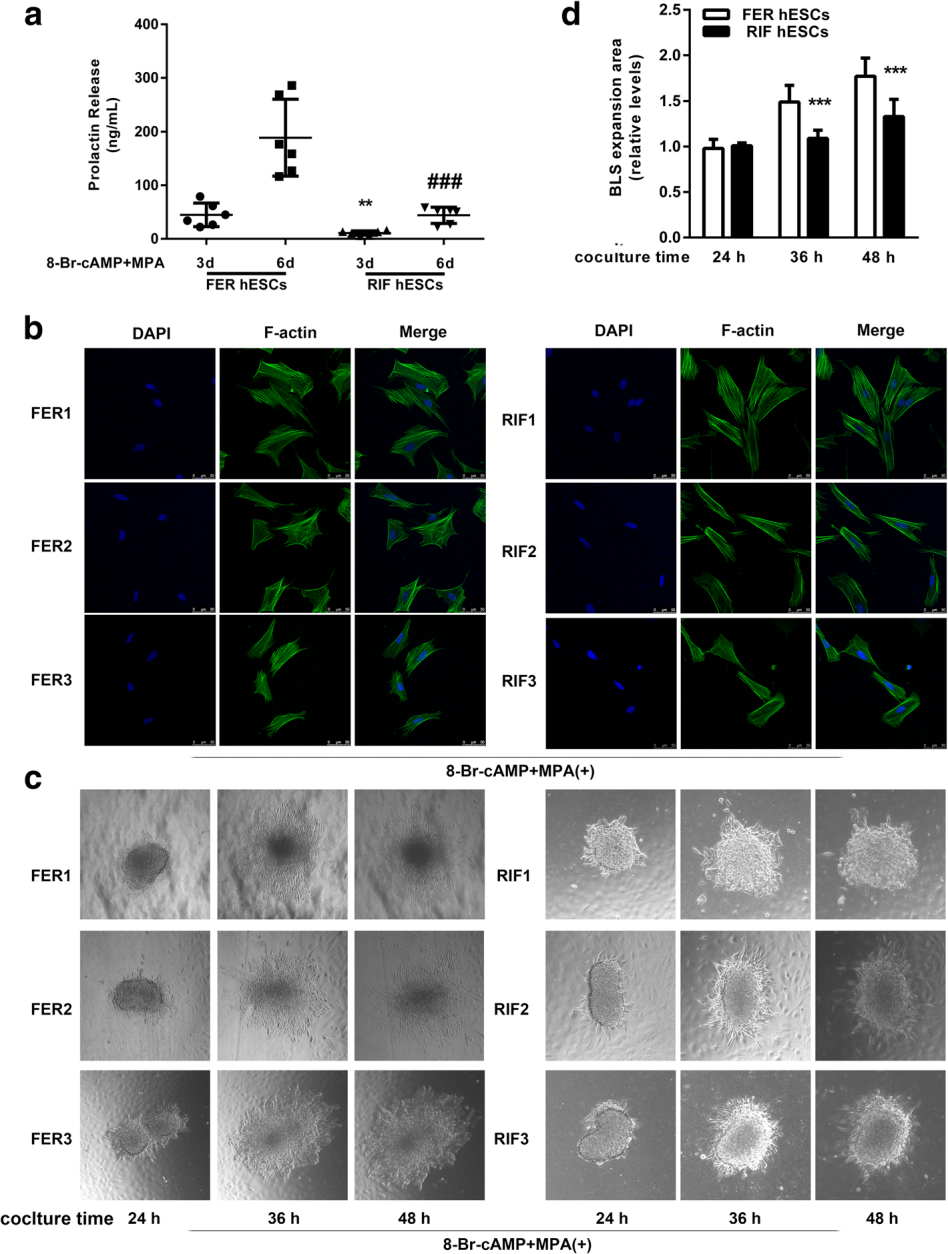
Impaired decidualization of hESCs isolated from endometrium of patients with RIF. **a** hESCs isolated from both RIF patients and fertile controls were treated with 0.5 mM 8-Br-cAMP and 1 μM MPA as indicated for an additional 3 days or 6 days. dPRL released into the medium was detected by ELFA. ^**^ *P* < 0.01 compared with fertile hESCs after treatment for 3 d; ^*###*^ *P < 0.001* compared with fertile hESCs after treatment for 6 d, repeated measures data ANOVA. **b** Fluorescein isothiocyanate-labeled phalloidin was used to label actin filaments, and immunofluorescence was adopted to analyze the morphological transformation of hESCs from both fertile controls (*n* = 6) and RIF patients (*n* = 6) after treatment with 0.5 mM 8-Br-cAMP and 1 μM MPA for 3 days. **c** Attachment of BLSs to hESC monolayers isolated from both RIF patients (*n* = 6) and fertile controls (*n* = 6) were assessed following treatment with 0.5 mM 8-Br-cAMP and 1 μM MPA as indicated for an additional 72 h. Several spheroids were transferred to confluent hESC monolayers from different women. Cells were imaged after 24 h, 36 h, and 48 h. **d** The area of BLS expansion on the FER hESCs and RIF hESCs were detected at different coculture time points and is presented relative to that of the 24 h time point (area was set to 1). ^***^ *P < 0.001* compared with FER hESCs group at the same time point, repeated measures data ANOVA

To confirm the poor decidual response of the hESCs from the endometrium of the RIF patients, we detected the previously described marker of decidualization. As shown in Fig. 1a, we observed that the secretion of dPRL was significantly reduced by approximately 75% in hESCs from the RIF patients compared with those from the fertile controls at both 3 days (10.84 ± 4.14 ng/ml versus 44.75 ± 22.00 ng/ml, *n* = 6, *P* < 0.01) and 6 days (44.12 ± 15.14 ng/ml versus 188.95 ± 71.89 ng/ml, *n* = 6, *P* < 0.001) after treatment with 8-Br-cAMP plus MPA. In addition, decidualized hESCs from the RIF patients showed an undifferentiated fibroblastic phenotype with a poorly formed actin cytoskeleton at 3 days after treatment with 8-Br-cAMP plus MPA (Fig. 1b).

To further assess the ability of the endometrium to accept embryo invasion, we used a previously published in vitro model of trophoblast spreading because of the importance of decidualization in the maintenance of pregnancy [23]. As demonstrated in Fig. 1c, BLSs attached to hESCs, and by 24 h, the BLSs moved forward between stromal cells, but this movement was limited in the RIF patients compared with the fertile controls. Moreover, we precisely located and measured the areas of BLS outgrowth at different time points and normalized these values to the initial area of BLS outgrowth at 24 h (Fig. 1d). The area of BLS expansion was significantly decreased by approximately 25% in the hESCs from the RIF patients compared with those from the fertile controls (36 h: 1.09 ± 0.09-fold versus 1.49 ± 0.18-fold; *n* = 6, *P* < 0.001, 48 h: 1.33 ± 0.19-fold versus 1.77 ± 0.20-fold; *n* = 6, *P* < 0.001).

### Aberrant expression of KLF12 and Nur77 in endometrium of patients with RIF

**Fig. 2:**
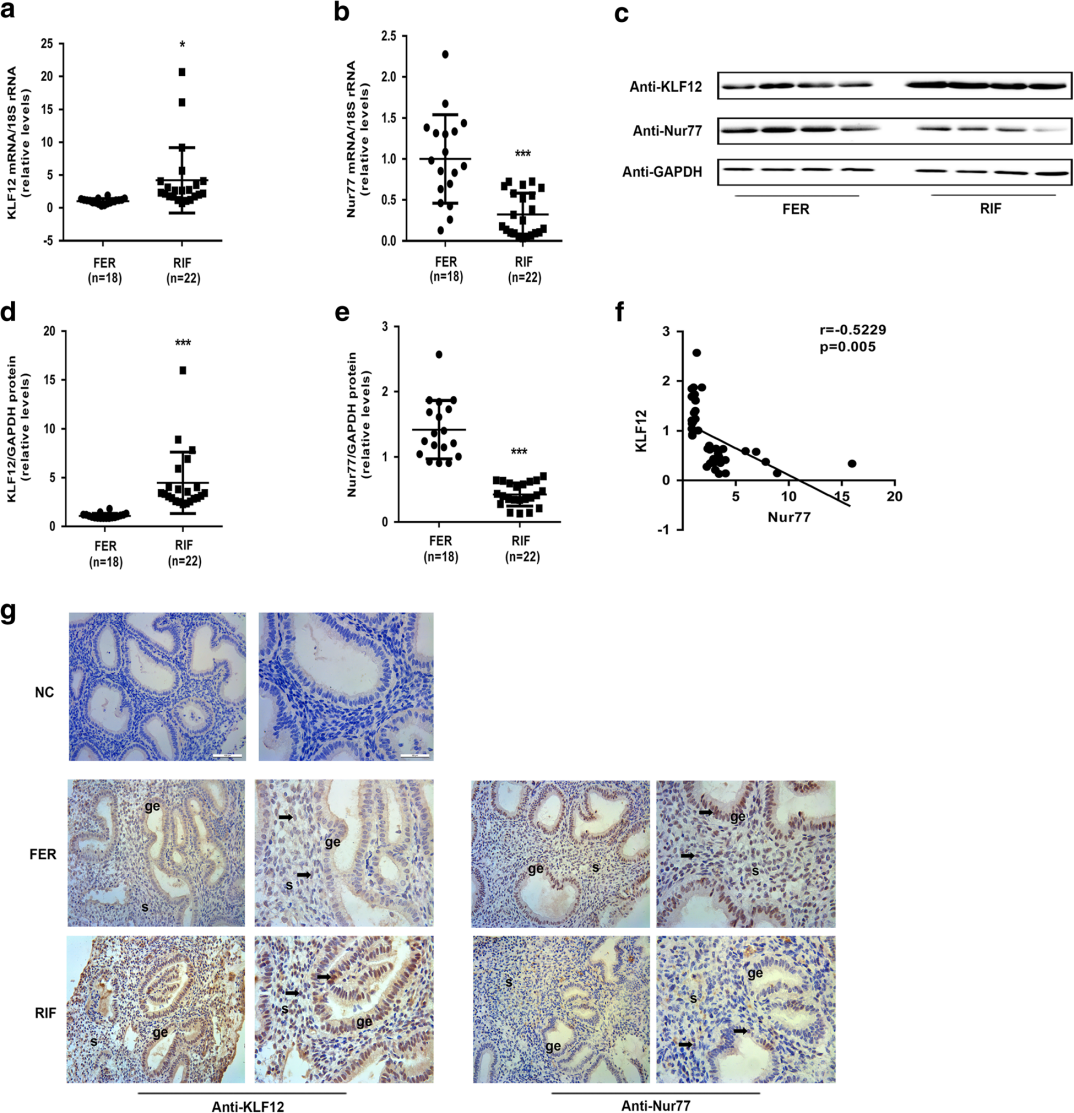
Aberrant expression of KLF12 and Nur77 in the endometrium of patients with RIF. **a** and **b** The KLF12 and Nur77 transcript levels in the endometrium of fertile (*n* = 18) and RIF (*n* = 22) patients were quantified by real-time PCR and were then normalized to control 18S gene expression. The data are presented relative to the fertile control group (expression was set to 1). ^*^ *P < 0.05* and ^***^ *P < 0.001* compared with the fertile group, Student’s t-test. **c** Differences in protein expression in endometrial samples were assessed by Western blotting using antibodies specific to KLF12 and Nur77. **d** and **e** The total KLF12 and Nur77 protein levels were normalized to GAPDH expression, and the data for all of the endometrial samples are shown in the scatter plots. ^***^ *P < 0.001* compared with the fertile group, Student’s t-test. **f** Correlation between KLF12 and Nur77 protein expression (*n* = 40, r = −0.5229, *P* = 0.005, Pearson correlation analysis). **g** Immunohistochemical analysis was performed using KLF12 and Nur77 antibodies. Endometrial tissue samples from fertile women and RIF patients are shown at 200× (left panel) and 400× (right panel) magnification. The negative control (NC) is nonspecific rabbit serum. Brown represents positive staining (arrows). Scale bars, 100 μm (left panel) and 50 μm (right panel)

Considering the aberrant decidualization of the hESCs from the RIF patients, we postulated that some decidual regulators could be expressed at altered levels in the endometrium of the RIF patients. The endometrial KLF12 mRNA and protein levels were markedly increased (by greater than 4-fold) during the implantation window in the RIF patients compared with the control women (Fig. 2a, c and d, mRNA: *P < 0.05*, protein: *P < 0.001*). In contrast, as expected, Nur77 expression was significantly reduced by 60% in the endometrium of the RIF patients (Fig. 2b, c and e, mRNA: *P < 0.001*, protein: *P < 0.001*). In addition, as shown in Fig. 2f, the protein levels of these two factors were moderately negatively correlated (r = −0.5229, *P* = 0.005). Furthermore, as demonstrated in immunolocalization analysis, the KLF12 protein abundance was greater and the Nur77 protein abundance was lower in the endometrium of the women with RIF compared with that of the fertile controls, especially in the endometrial stroma in the RIF tissue samples (Fig. 2g).

### Nur77 is a novel target gene of KLF12 in hESCs

**Fig. 3:**
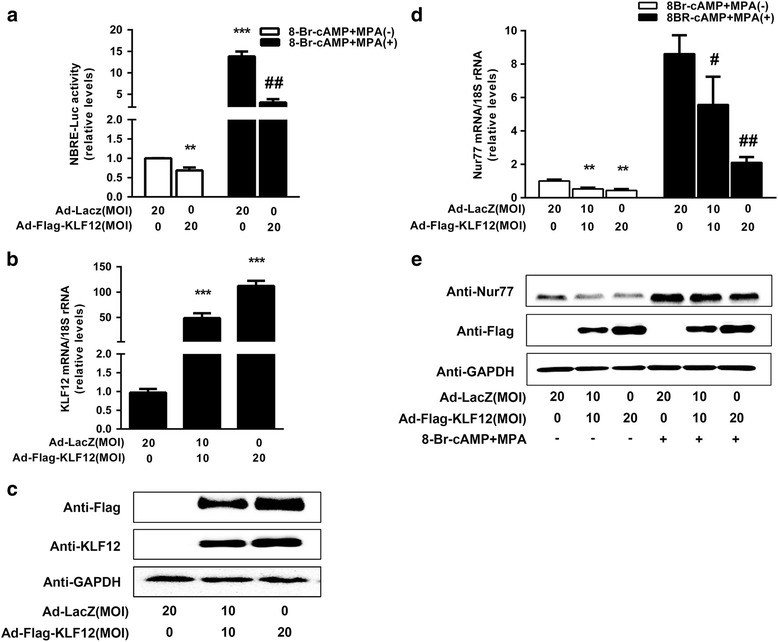
Enhanced KLF12 expression led to a decrease in Nur77 expression. **a** hESCs were infected with the indicated adenoviruses for 24 h, transfected with NBRE-Luc (300 ng/well), and then treated with 8-Br-cAMP and MPA. After 48 h, luciferase assays were performed, and the data were plotted after normalization to Renilla luciferase activity. ^**^ *P < 0.01* and ^***^ *P < 0.001* compared with the Ad-LacZ without 8-Br-cAMP and MPA group; ^*##*^ *P < 0.01* compared with the Ad-LacZ with 8-Br-cAMP and MPA group, ANOVA followed by a Student–Newman–Keuls test. (**b**) and (**c**) hESCs were infected with an adenovirus expressing either LacZ or KLF12 for 48 h at the indicated MOI. The KLF12 transcript copy number and protein expression were determined by real-time PCR and Western blotting. ^***^ *P < 0.001* compared with the Ad-LacZ only-treated group, ANOVA followed by a Student–Newman–Keuls test. (**d**) and (**e**) hESCs were infected with Ad-LacZ or Ad-Flag-KLF12 at the indicated MOI for 48 h and then treated with 8-Br-cAMP and MPA for another 2 or 3 days; subsequently, the Nur77 mRNA and protein levels were measured by real-time PCR and Western blotting, respectively. ^**^ *P < 0.01* compared with Ad-LacZ alone; ^*#*^ *P < 0.05* and ^*##*^ *P < 0.01* compared with the Ad-LacZ plus 8-Br-cAMP and MPA group, ANOVA followed by a Student–Newman–Keuls test

**Fig. 4:**
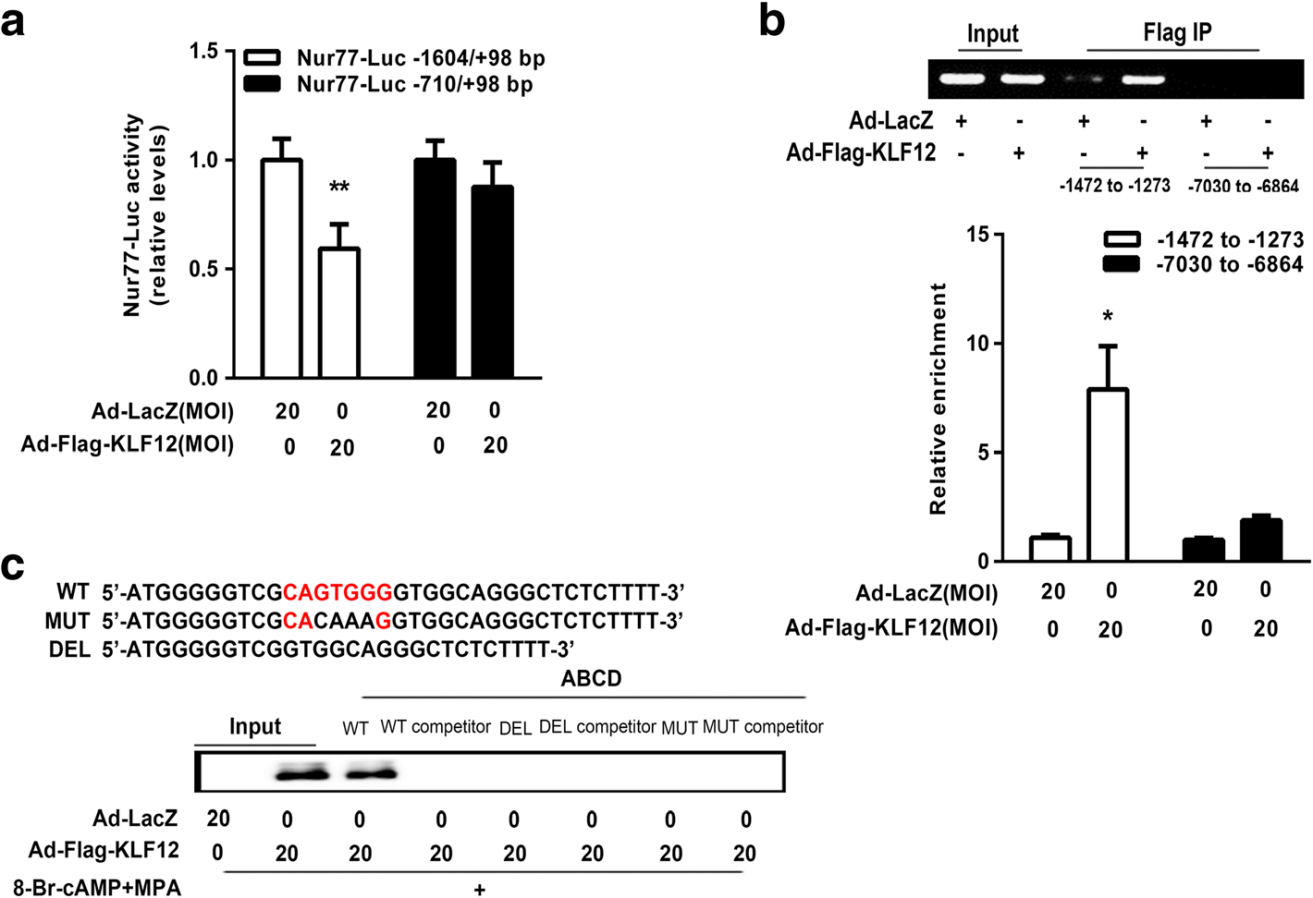
KLF12 directly repressed Nur77 transcription. **a** hESCs were infected with the indicated adenoviruses for 24 h and then transfected with Nur77-Luc (−1604/+98 bp) or deleted Nur77-Luc (−710/+98 bp) (300 ng/well). After 48 h, luciferase assays were performed, and the data were plotted after normalization to Renilla luciferase activity. ^**^ *P < 0.05* compared with Ad-LacZ alone, Student’s t-test. **b** ChIP-PCR amplification using primers against the human Nur77 promoter region (top). PCR products were separated by agarose gel electrophoresis. Quantitative ChIP analysis was performed by real-time PCR. The results are shown as fold changes relative to LacZ (after normalization to the input DNA, bottom). Input (non-precipitated) chromatin was utilized as a positive control in these analyses. ^*^ *P < 0.05* compared with Ad-LacZ alone, Student’s t-test. **c** ABCD assays were performed using biotinylated or non-biotinylated (competitor) double-stranded Nur77 wild-type (WT), conserved element-deleted (DEL) and conserved element-mutated (MUT) oligonucleotides with whole-cell extracts from hESCs

Because the Nur77 core promoter region contains a KLF12-binding element, we suspected that Nur77 could be a novel target of KLF12. The activity of the NBRE (AAAGGTCA) element, which was elevated by Nur77 overexpression, was repressed in KLF12-overexpressing hESCs, as demonstrated by luciferase reporter assay (Fig. 3a, ^**^ *P* < 0.01 and ^***^ *P* < 0.001 compared with the Ad- LacZ without 8-Br-cAMP and MPA group; ^*##*^ *P < 0.01* compared with the Ad-LacZ with 8-Br-cAMP and MPA group). These findings indicated that KLF12 might regulate NR4A family protein expression. As observed in Fig. 3d and e, KLF12 overexpression in hESCs (Fig. 3a, b) resulted in significant dose-dependent decreases in both the Nur77 mRNA and protein abundance (^**^ *P* < 0.01 compared with Ad-LacZ alone; ^*#*^ *P* < 0.05 and ^*##*^ *P* < 0.01 compared with the Ad-LacZ plus 8-Br-cAMP and MPA group). In addition, the luciferase reporter assay results demonstrated that exogenous hormone-induced decidual transformation caused an increase in Nur77 promoter activity and that this increase could be repressed by KLF12 overexpression in hESCs (Fig. 4a, *P* < 0.01).

Next, conventional ChIP-PCR analysis was further conducted to investigate whether the Nur77 promoter is a direct target of KLF12 in hESCs. As shown in Fig. 4b, this promoter (−1472 to −1273 bp) was effectively recovered from immunoprecipitates of the Flag-KLF12 protein, but it was not recovered from those of the LacZ control. In addition, no PCR product was obtained from the Flag-KLF12 or LacZ control immunoprecipitate using the negative control primers (−7030 to −6864 bp, Fig. 4b). Moreover, quantitative ChIP-PCR revealed that the primers effectively (greater than 8-fold) amplified their targets in the Flag-KLF12 protein immunoprecipitates but not in the LacZ control immunoprecipitates (Fig. 4b, *P* < 0.05). In addition, ABCD assays were performed using biotinylated double-stranded oligonucleotides corresponding to the WT (−1391/−1357 bp), deleted (DEL) and mutated (MUT) Nur77 promoter sequences. The results showed that the Flag-tagged KLF12 proteins strongly bound to the WT probe but not to the DEL or MUT probe (Fig. 4c). Taken together, these results demonstrated that KLF12 transcriptionally repressed Nur77 by directly binding to the conserved CAGTGGG element in the promoter region.

### KLF12 impaired hESC decidualization in vitro by repressing Nur77 expression

**Fig. 5:**
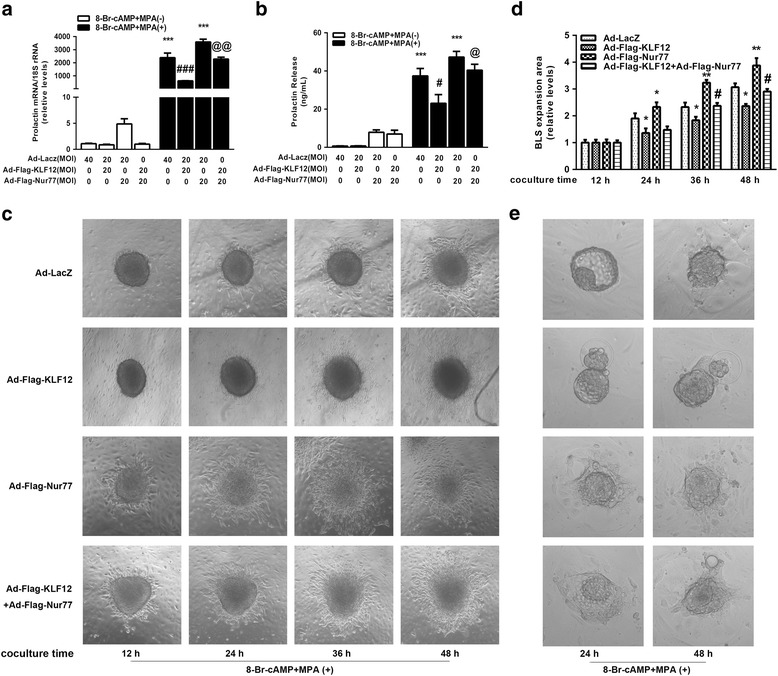
KLF12 impaired hESC decidualization in vitro by repressing Nur77 expression. (**a**) and (**b**) hESCs were infected with Ad-Flag-KLF12, Ad-Flag-Nur77 or Ad-LacZ (MOI = 20). After 24 h, the cells were treated with 0.5 mM 8-Br-cAMP and 1 μM MPA as indicated for an additional 72 h. dPRL released into the medium was detected by ELFA. dPRL mRNA levels were measured by real-time PCR. ^***^ *P < 0.01* compared with the 8-Br-cAMP + MPA (−) group; ^*#*^ *P < 0.05* and ^*###*^ *P < 0.001* compared with the 8-Br-cAMP + MPA (+) group; and ^*@*^ *P < 0.05* and ^*@@*^ *P < 0.01* compared with the Ad-Flag-KLF12 (MOI = 20) plus 8Br-cAMP + MPA group, ANOVA followed by a Student–Newman–Keuls test. (**c**) and (**d**) Attachment of BLSs and their expansion onto hESC monolayers. hESCs were infected with Ad-Flag-KLF12, Ad-Flag-Nur77 or Ad-LacZ (MOI = 20). After 24 h, the cells were treated with 0.5 mM 8-Br-cAMP and 1 μM MPA as indicated for an additional 72 h. Several spheroids were transferred to confluent monolayers of hESCs subjected to the different treatments. Cells were imaged after 12 h, 24 h, 36 h, and 48 h. The area of BLS expansion on the hESCs was detected at different time points and is presented relative to that of the Ad-LacZ group (area was set to 1). ^*^ *P < 0.05* and ^**^ *P < 0.01* compared with Ad-LacZ group at the same time point; ^*#*^ *P < 0.05* compared with Ad-Flag-KLF12 group at the same time point, repeated measures data ANOVA. **e** Analysis of blastocyst attachment and expansion. hESCs were infected with Ad-Flag-KLF12, Ad-Flag-Nur77 or Ad-LacZ (MOI = 20). After 24 h, the cells were treated with 0.5 mM 8-Br-cAMP and 1 μM MPA for an additional 72 h. Blastocysts were collected from the uterus of a pregnant mouse in the afternoon at 3.5 days post-coitus (dpc). Several blastocysts were transferred to confluent monolayers of hESCs subjected to the different treatments. Blastocysts were imaged after 24 h and 48 h

As Nur77 was determined to be a novel target of KLF12, we further investigated whether Nur77 is able to reverse the impaired decidualization induced by KLF12. KLF12-enhanced hESCs were subjected to adenovirus-mediated overexpression of Nur77 and treated with 8-Br-cAMP plus MPA to determine the vital role of Nur77. As shown in Fig. 5a  and b, the transcript and secretion levels of dPRL were increased following Nur77 overexpression compared with those observed following treatment with 8-Br-cAMP plus MPA alone (23.09 ± 4.53 ng/mL versus 37.43 ± 3.88 ng/mL, Ad-Flag-KLF12 with 8-Br-cAMP + MPA (+) versus 8-Br-cAMP + MPA (+) alone, ^*#*^ *P* < 0.05; 40.44 ± 3.09 ng/mL versus 23.09 ± 4.53 ng/mL, Ad-Flag-Nur77+ Ad-Flag-KLF12 with 8-Br-cAMP + MPA (+) versus Ad-Flag-KLF12 with 8-Br-cAMP + MPA (+), ^*@*^ *P < 0.05*). In addition, KLF12 overexpression caused the decidualized hESCs to acquire long, fibroblast-like shape. Further, Nur77 overexpression caused the hESCs to become noticeably rounder and caused the disarrangement of actin filaments (Additional file 1: Figure S1).

Moreover, the BLS implantation model was conducted to confirm the complementary function of Nur77. As shown in Fig. 5c and d, the area of BLS expansion was reduced by approximately 25% in KLF12-enhanced decidual hESCs at 24 h, 36 h and 48 h, and it could be enhanced to a certain extent compared with that in the control cells by overexpressing Nur77 (^*^ *P* < 0.05 and ^**^ *P* < 0.01 compared with Ad-LacZ group at the same time point; ^*#*^ *P* < 0.05 compared with Ad-Flag-KLF12 group at the same time point). Similar findings were observed in mouse blastocyst implantation model (Fig. 5e).

### Poor decidual response of hESCs from RIF patients was reversed by increasing Nur77 expression

**Fig. 6:**
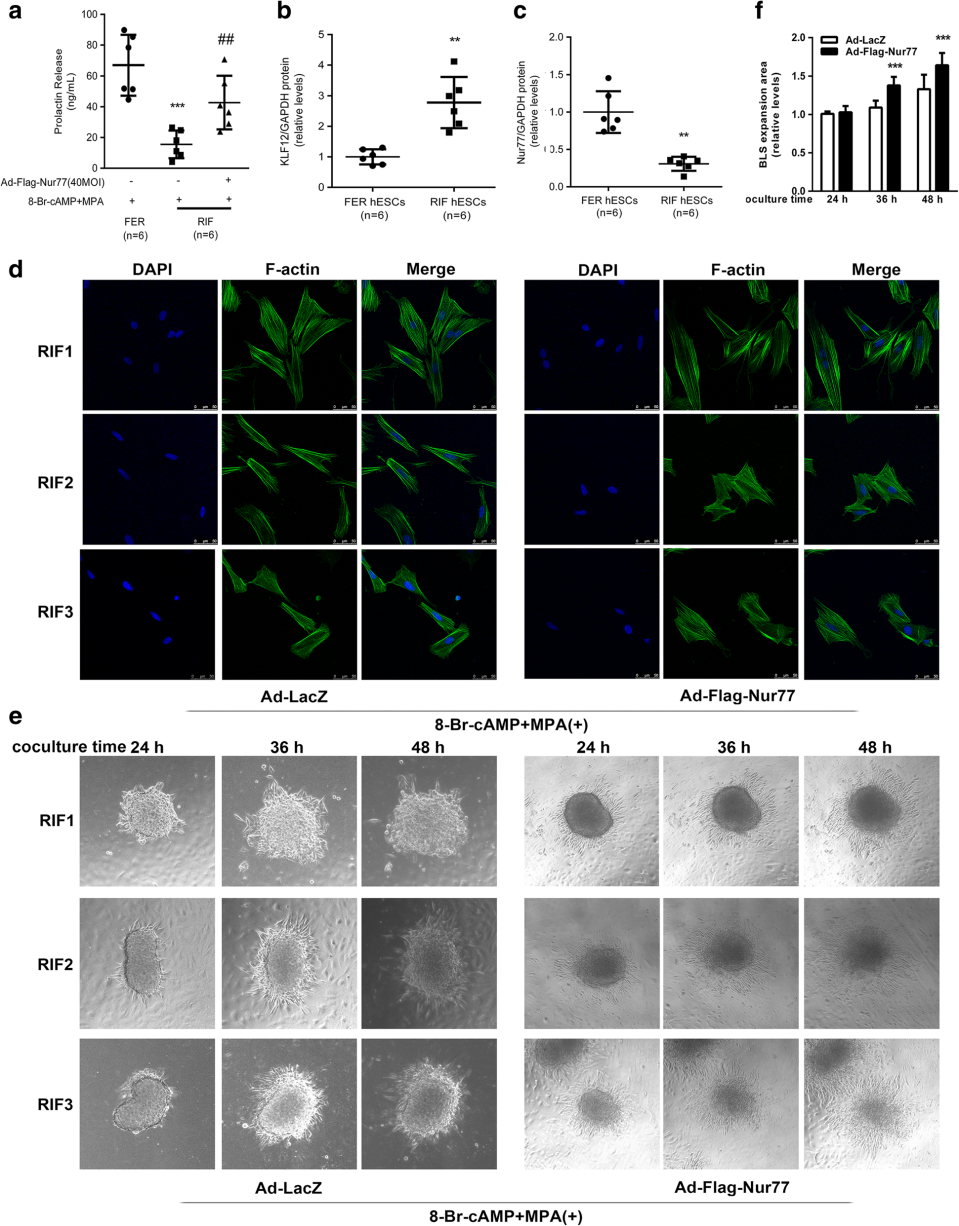
Poor decidual response of hESCs from RIF patients was reversed by increasing Nur77 expression. **a** hESCs from RIF patients were infected with Ad-Flag-Nur77 or Ad-LacZ (MOI = 20). After 24 h, the cells were treated with 0.5 mM 8-Br-cAMP and 1 μM MPA as indicated for an additional 3 days. dPRL released into the medium was detected by ELFA. ^***^ *P < 0.001* compared with fertile controls; ^*##*^ *P < 0.01* compared with RIF hESCs with Ad-LacZ, ANOVA followed by a Student–Newman–Keuls test. (**b**) and (**c**) The KLF12 and Nur77 protein levels in hESCs from both fertile women (*n* = 6) and RIF patients (*n* = 6) were detected by Western blotting. ^**^ *P < 0.01* compared with fertile controls. **d** Fluorescein isothiocyanate-labeled phalloidin was used to label actin filaments, and immunofluorescence was used to analyze the morphological transformation of hESCs from RIF patients treated with 0.5 mM 8-Br-cAMP and 1 μM MPA for 3 days after infection with Ad-Flag-Nur77 or Ad-LacZ (MOI = 20). **e** Attachment of BLSs and their expansion onto hESCs monolayers. hESCs from both fertile women (*n* = 6) and RIF patients (*n* = 6) were infected with Ad-Flag-Nur77 or Ad-LacZ (MOI = 20). After 24 h, the cells were treated with 0.5 mM 8-Br-cAMP and 1 μM MPA as indicated for an additional 72 h. Several spheroids were transferred to confluent monolayers of hESCs subjected to the different treatments. Cells were imaged after 24 h, 36 h, and 48 h. **f** The area of BLS expansion on the RIF hESCs infected with Ad-Flag-Nur77 or Ad-LacZ (MOI = 20) were detected at different time points and is presented relative to that of the Ad-LacZ group (area was set to 1). ^*^ *P < 0.001* compared with Ad-LacZ group at the same time point, repeated measures data ANOVA

Considering our finding that Nur77 overexpression rescued the impaired decidual response induced by exogenous KLF12 overexpression in vitro, the function of Nur77 in protecting against pathological decidualization in RIF patients was further investigated. The results suggested that KLF12 expression was higher in hESCs from the women with RIF (*n* = 6) than in those from the fertile controls (*n* = 6) (Fig. 6b, *P* < 0.01). In contrast, Nur77 expression was significantly decreased (Fig. 6c, *P* < 0.01). The decreased dPRL secretion in decidual hESCs treated with 8-Br-cAMP and MPA from the RIF patients could be enhanced to a level similar to that observed in the fertile controls by overexpressing Nur77 (Fig. 6a, ^***^ *P* < 0.001 compared with fertile controls; ^*##*^ *P* < 0.01 compared with RIF hESCs with Ad-LacZ). Moreover, a function of Nur77 in improving disrupted decidualization was observed by F-actin immunofluorescence (Fig. 6d) and BLS implantation model (Fig. 6e, f, 36 h: 1.38 ± 0.11-fold versus 1.09 ± 0.09-fold; *n* = 6, *P* < 0.001, 48 h: 1.64 ± 0.16-fold versus 1.33 ± 0.19-fold; *n* = 6, *P* < 0.001). Taken together, these results demonstrated that Nur77 overexpression reversed the poor decidual response in the RIF hESCs caused by increased KLF12 expression.

## Discussion

Pregnancy is a complex process characterized by discrete events, including implantation, placentation, and finally, the birth of offspring. Implantation, a vital process, is controlled by a sophisticated interaction between the embryo and endometrium [26–29]. Despite the improvements in IVF technology achieved in recent years, inadequate endometrial receptivity and impaired decidualization are considered the major limiting factors for the success of quality embryo implantation [30, 31]. This study is the first to demonstrate that dysregulated KLF12 expression in the endometrium of RIF patients results in repression of Nur77 transcription, leading to impaired decidualization and ultimately to implantation failure.

Krüppel-like factor 12 is a member of the Krüppel-like factor (KLF) family of zinc-finger transcription factors that are critical regulators of cell differentiation, phenotypic modulation, and physiologic function [32]. The KLF family plays multiple roles in embryo implantation. Mice null for KLF5 or the progesterone receptor (PGR)-interacting protein KLF9 are subfertile, as KLF5 is critical for the establishment of uterine decidualization [33]. On the other hand, cross-regulation between KLF9/KLF13 and BMP2 is essential for the maintenance of progesterone sensitivity in differentiating stromal cells [34, 35]. In addition*,* KLF15 is a hormone-related gene that blocks Ishikawa cell proliferation by binding to the Mcm2 promoter [36]. Further, KLF12, a transcription factor that binds to the promoter regions of target genes and represses their expression through an N-terminal PVDLS sequence (Pro-Xaa-Asp-Leu-Ser), recognizes and interacts with the CAGTGGG sequence [37, 38]. The results of this study showed that KLF12 bound to a specific site in the Nur77 promoter region, negatively affecting decidualization and leading to embryo implantation failure. Moreover, increasing Nur77 expression rescued the KLF12-induced poor decidual response by increasing the secretion of dPRL, restoring the cytoskeletal structure and enhancing embryo expansion.

From research to the clinic, Nur77 has been reported to be an important factor promoting the up-regulation of dPRL expression in a process partly mediated by FOXO1A. Furthermore, Nur77 has been reported to be an activator of decidualization that rescues impaired decidualization in adenomyosis [14]. Similarly, in this study, we observed that Nur77 reversed the decreased dPRL secretion in RIF hESCs. Moreover, BLS and blastocyst implantation models were conducted to provide precise, visible evidence of the complementary function of Nur77 in the impaired decidualization caused by KLF12 in RIF patients. Thus, the identification of a positive agonist of Nur77 will be beneficial to the improvement of treatments for RIF patients with conditions involving insufficient decidualization.

In this study, we also quantified the relative abundance of KLF12 and Nur77 in hESCs following treatment with 8-Br-cAMP and MPA (data not shown). The repression of KLF12 expression was observed by 48 h after treatment. In contrast, Nur77 expression was rapidly induced after in vitro decidual stimulation. These findings suggest that KLF12 functions as a novel and critical ‘on-off’ switch during decidualization. The orphan nuclear eceptor Nur77, a member of the NR4A receptor family of ligand-independent transcription factors and immediate- and early-response genes, is usually rapidly induced by various environmental cues [39]. It might only function during the initiation of decidualization. In the endometrium of the women with RIF, the enhanced KLF12 expression led to a reduction in Nur77 expression, which resulted in the repression of early decidual activation. However, the continuous high expression of KLF12 in the endometrium of RIF patients could also result in disruption of the maintenance of decidualization, which is normally maintained via a decreased KLF12 level. Throughout pregnancy, the decidua usually forms a dense cellular matrix that generates a local cytokine environment, thereby promoting trophoblast attachment while limiting aggressive invasion by fetal tissues [40, 41]. Trophoblast invasion requires proteolytic degradation and remodeling of the decidual matrix. The process of decidualization is necessary for decidual matrix formation. Therefore, the impaired decidualization caused by enhanced KLF12 expression leads to limited BLS and blastocyst expansion. Embryos secrete several matrix metalloproteinases (MMPs) to facilitate their expansion and invasion into decidual hESCs [42]. The actions of MMPs are opposed by tissue inhibitors of metalloproteinases (TIMPs), which are produced both by trophoblast cells themselves and by decidual cells [43, 44]. On the other hand, Nur77 has been reported to play important roles in promoting cancer cell invasion, metastasis and vascular remodeling by regulating MMPs and TIMPs [45–47]. Thus, whether the regulation of MMPs and TIMPs by Nur77 plays a vital role in embryo expansion and invasion should be further investigated. In addition, MMPs and TIMPs, such as MMP2, MMP3, MMP9, TIMP1 and TIMP3, should be detected in KLF12-overexpressing hESCs after 8-Br-cAMP and MPA treatment to determine the function of KLF12 in trophoblast invasion and throughout pregnancy.

Although we identified the functions of KLF12 and Nur77 in the decidualization of hESCs from RIF patients, the immunohistochemical results revealed that KLF12 expression was increased not only in the stromal compartment but also in the GE. The opposite findings were observed for Nur77 protein expression. Implantation failure may occur very early during the attachment stage or later on following successful migration of the embryo through the luminal surface [19]. The abnormal expression of KLF12 and Nur77 in the GE could decrease receptivity of the endometrium in RIF patients, which would result in the first type of implantation failure. Therefore, the exact mechanism of KLF12 and Nur77 regulation in the GE should be further assessed. On the other hand, to prevent exogenous adenovirus-mediated enhanced KLF12 expression in vitro, a mouse model with a uterine conditional KLF12 knock-in will be employed in a future study to assess the physiological function of KLF12 and compensatory function of Nur77 in embryo implantation.

This study is the first to functionally analyze the role of Nur77 in reversing the poor decidual response of KLF12 pathologically enhanced hESCs from RIF patients. In addition, our results have revealed a new potential mechanism of impaired decidualization that may be relevant in patients with implantation failure occurring after detection of hCG in the blood, which is clinically referred to as a biochemical pregnancy.

## Conclusions

Taken together, our results indicate that aberrantly increased KLF12, by negatively regulating Nur77 expression, contributes to improper stromal decidualization and reduced embryo implantation in RIF patients. These findings may provide novel potential therapeutic regimens for patients with RIF and disrupted decidualization.

## Additional files

Additional file 1: Figure S1. Decidual transformation change of hESCs treated with Ad-Flag-KLF12 or Ad-Flag-Nur77. Fluorescein isothiocyanate-labeled phalloidin was used to label actin filaments, and immunofluorescence was used to analyze the morphological transformation of hESCs treated with 0.5 mM 8-Br-cAMP and 1 μM MPA for 3 days after infected with Ad-Flag-KLF12, Ad-Flag-Nur77 or Ad-LacZ (MOI = 20). (TIF 1183 kb)

Additional file 2: Editorial Certificate of American Journal Experts. (PDF 909 kb)

Additional file 3: The original IRB approval. (DOC 155 kb)

Additional file 4: English translation of the IRB approval. (DOC 64 kb)

## Abbreviations

ABCD: Avidin-biotin conjugate DNA precipitation
ART: Assisted reproductive technology
BLS: Blastocyst-like spheroid
ChIP/PCR: Chromatin immunoprecipitation PCR
Dpc: Days post-coitus
dPRL: Decidual prolactin
ELFA: Enzyme-linked fluorescent assay
GE: Glandular epithelium
hESCs: Human endometrial stromal cells
IVF: In vitro fertilization
KLF12: Krüppel-like factor 12
MMP: Matrix metalloproteinase
MPA: Medroxyprogesterone-acetate
qRT-PCR: Real-time-quantitative PCR
RIF: Recurrent implantation failure
RIF: Recurrent implantation failure
RPL: Recurrent pregnancy loss
TIMP: Tissue inhibitors of metalloproteinase
