# Supplementary figures and images for: Increased Krüppel-like factor 12 in recurrent implantation failure impairs endometrial decidualization by repressing Nur77 expression
Source: Reprod Biol Endocrinol. 2017 Mar 31;15:25. doi: 10.1186/s12958-017-0243-8 (PMC5374626; doi:10.1186/s12958-017-0243-8)

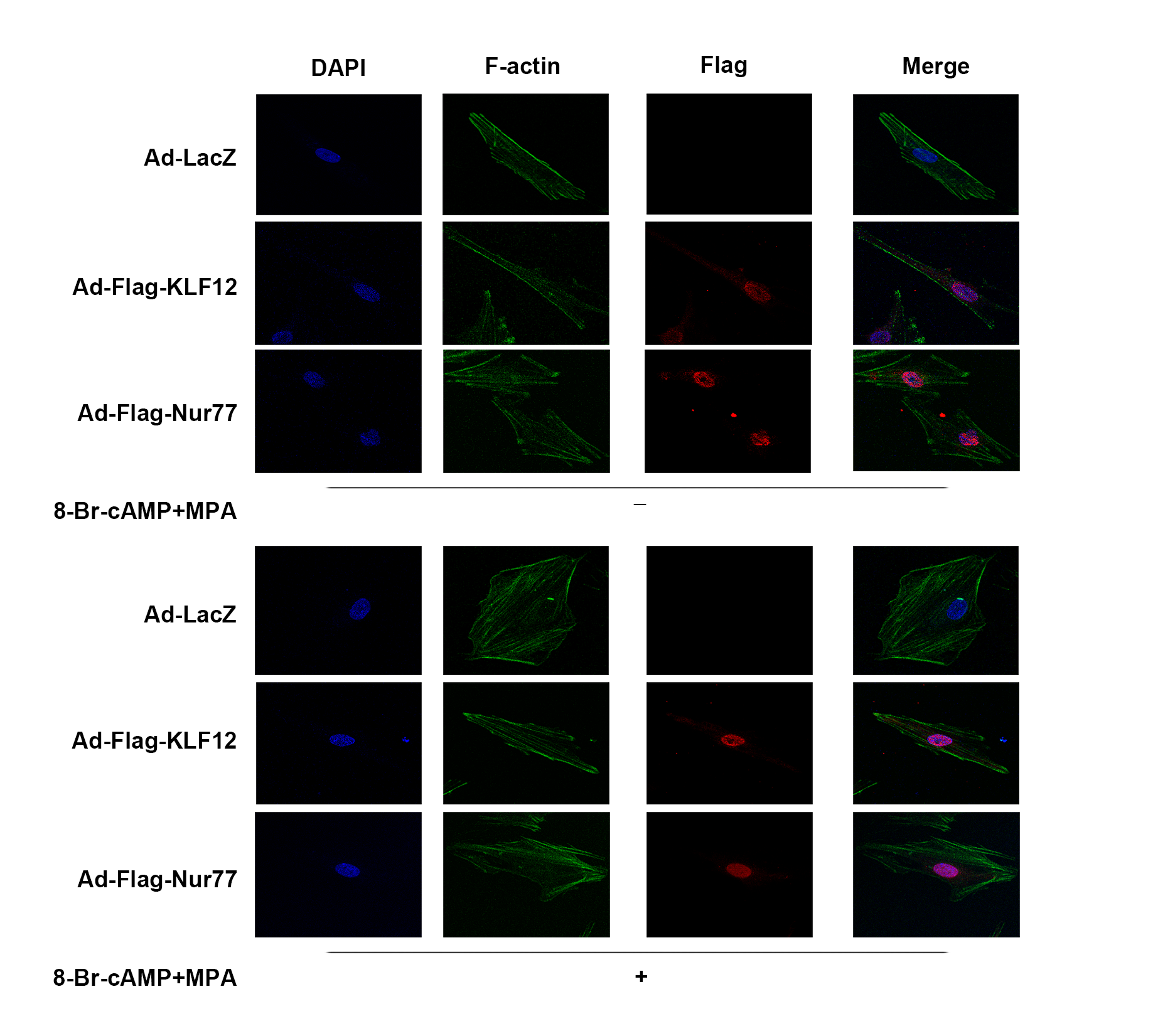

Supplement: Supplementary file 1 — Decidual transformation change of hESCs treated with Ad-Flag-KLF12 or Ad-Flag-Nur77. Fluorescein isothiocyanate-labeled phalloidin was used to label actin filaments, and immunofluorescence was used to analyze the morphological transformation of hESCs treated with 0.5 mM 8-Br-cAMP and 1 μM MPA for 3 days after infected with Ad-Flag-KLF12, Ad-Flag-Nur77 or Ad-LacZ (MOI = 20). (TIF 1183 kb) [file 12958_2017_243_MOESM1_ESM.tif]

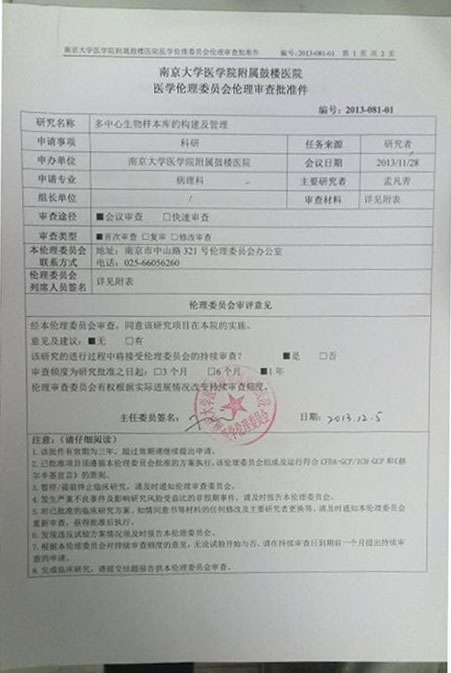


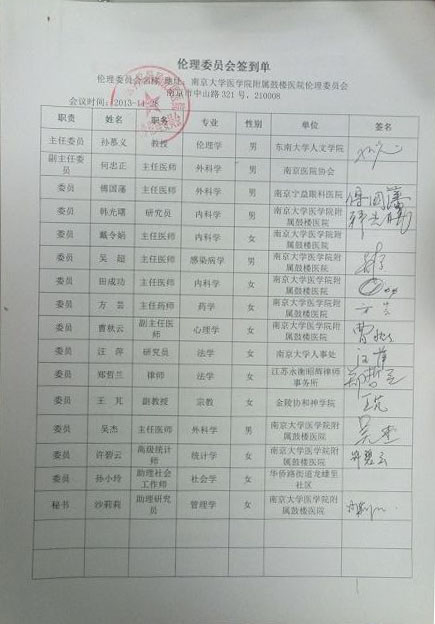

Supplement: Supplementary file 3 — The original IRB approval. (DOC 155 kb) [file 12958_2017_243_MOESM3_ESM.doc]
