# Supplementary material for: Increased Krüppel-like factor 12 in recurrent implantation failure impairs endometrial decidualization by repressing Nur77 expression
Source: Reprod Biol Endocrinol. 2017 Mar 31;15:25. doi: 10.1186/s12958-017-0243-8 (PMC5374626; doi:10.1186/s12958-017-0243-8)
Supplement: Supplementary file 2 — Editorial Certificate of American Journal Experts. (PDF 909 kb) [file 12958_2017_243_MOESM2_ESM.pdf]

AMERICAN JOURNAL EXPERTS

EDITORIAL CERTIFICATE

This document certifies that the manuscript listed below was edited for proper English language, grammar, punctuation, spelling, and overall style by one or more of the highly qualified native English speaking editors at American Journal Experts.

Manuscript title:

Increased Krüppel-like factor 12 in recurrent implantation failure impairs endometrial decidualization through repressing Nur77 expression

Authors:

Chenyang Huang, Yue Jiang, Jianjun Zhou, Qiang Yan, Ruiwei Jiang, Xi Cheng, Jun Xing, Lijun Ding, Jianxin Sun, Guijun Yan, Haixiang Sun

Date Issued:

November 4, 2016

Certificate Verification Key:

0BB1-7EAB-CD37-90A7-DF6C

This certificate may be verified at www.aje.com/certificate. This document certifies that the manuscript listed above was edited for proper English language, grammar, punctuation, spelling, and overall style by one or more of the highly qualified native English speaking editors at American Journal Experts. Neither the research content nor the authors' intentions were altered in any way during the editing process. Documents receiving this certification should be English-ready for publication; however, the author has the ability to accept or reject our suggestions and changes. To verify the final AJE edited version, please visit our verification page. If you have any questions or concerns about this edited document, please contact American Journal Experts at support@aje.com.

American Journal Experts provides a range of editing, translation and manuscript services for researchers and publishers around the world. Our top-quality PhD editors are all native English speakers from America's top universities. Our editors come from nearly every research field and possess the highest qualifications to edit research manuscripts written by non-native English speakers. For more information about our company, services and partner discounts, please visit www.aje.com.
