# Supplementary material for: Increased Krüppel-like factor 12 in recurrent implantation failure impairs endometrial decidualization by repressing Nur77 expression
Source: Reprod Biol Endocrinol. 2017 Mar 31;15:25. doi: 10.1186/s12958-017-0243-8 (PMC5374626; doi:10.1186/s12958-017-0243-8)
Supplement: Supplementary file 4 — English translation of the IRB approval. (DOC 64 kb) [file 12958_2017_243_MOESM4_ESM.doc]

**The ethical review approval from the IRB of the Drum Tower Hospital of Nanjing University**

**No: 2013-081-01**

| Project name | Construction and management of the Nanjing multi-center biobank | | |
| --- | --- | --- | --- |
| Purpose | Scientific research | Source | researcher |
| Unit | Drum Tower Hospotal of Nanjing University | Meeting date | Nov. 28, 2013 |
| Department | Pathology department | Principal investigator (PI) | Fanqing Meng |
| Department of PI | Pathology department | Material for review | Refer to the attachment |
| Methods for review | ■meeting review □expedited review | | |
| Type of review | ■initial review □continuing review □review after revision | | |
| Contact of the IRB | Address: No.321 Zhongshan Road, the IRB office of Nanjing, China  Tel: 025-66056266 | | |
| Signature of the IRB members | Refer to the attachment | | |
| Recommendation opinion from the IRB | | | |
| According to the IRB review decision, agreenment is made for this project.  Opinions and suggestions: ■No □Yes  Will the study be reviewed regularly? ■Yes □No  The frequency of continuing review: □3 months □6 months ■1 year  The IRB has the authority to change the frequency of continuing review.  Signature of committee chairman: Muyi Sun Date: Dec. 5, 2013 | | | |
| Attention | | | |

**IRB members of the Drum Tower Hospital of Nanjing University**

| **Position** | **Name** | **Technical post** | **Major** | **Gender** | **Administrative unit** |
| --- | --- | --- | --- | --- | --- |
| Chairman | Muyi Sun | Professor | Ethics | Male | School of Humanities Southeast University |
| Vice-chairman | Zhongzheng Ke | Chief Physician | Surgery | Male | Nanjing Hospital Association |
| Member | Guofan Fu | Chief Physician | Surgery | Male | Nanjing Yining Ophthalmic Hospital |
| Member | Guangshu Han | Professor | Internal medicine | Male | the Drum Tower Hospital of Nanjing University |
| Member | Lingjuan Dai | Chief Physician | Internal medicine | Female | the Drum Tower Hospital of Nanjing University |
| Member | Chao Wu | Chief Physician | Infectious diseases | Male | the Drum Tower Hospital of Nanjing University |
| Member | Chenggong Tian | Chief Physician | Internal medicine | Female | the Drum Tower Hospital of Nanjing University |
| Member | Yun Fang | Chief Physician | Pharmaceutical Science | Female | the Drum Tower Hospital of Nanjing University |
| Member | Qiuyun Cao | Associate Chief Physician | psychology | Female | the Drum Tower Hospital of Nanjing University |
| Member | Ping Wang | Professor | Law | Female | The personnel department of Nanjing University |
| Member | Zhelan Zheng | lawyer | Law | Female | Jiangsu Yong Heng Chao Hui law firm |
| Member | Qi Wang | Associate Professor | Religion | Female | Nanjing Theological Seminary |
| Member | Jie Wu | Chief Physician | Surgery | Male | the Drum Tower Hospital of Nanjing University |
| Member | Biyun Xu | Senior Statistician | Statistics | Female | the Drum Tower Hospital of Nanjing University |
| Member | Xiaoling Sun | Assistant social worker | Sociology | Female | Nanjing Huaqiao Rroad Longpanli Community |
| Member | Lili Sha | Research Associate | Management | Female | the Drum Tower Hospital of Nanjing University |
